# Society Meetings

**Published:** 1912-10

**Authors:** 


### SOCIETY MEETINGS.

New York and New England Association of Railway Surgeons.—The twenty-second annual session will be held at the Hotel Astor, New York City, on Wednesday, November 15. An interesting program has been arranged. Dr. John B. Murphy, of Chicago, will deliver the "Address in Surgery." Railway surgeons, attorneys and officials and all members of the medical profession are cordially invited to attend.

Dr. Walter Lathrop, President, Hazelton, Penna. Dr. George Chaffee, Corresponding Secretary, 338 Forty-seventh Street, Brooklyn, N. Y.

The Nurses' Alumnae Association of the Children's Hospital, Buffalo, at its meeting of Sept. 10, decided to incorporate. Meetings will be held on the second Tuesday of each month except July and August. Four of the meetings will be social, when the members of the training school will be entertained. Progress in medicine, surgery and nursing, will be considered at the other meetings.

The Buffalo Dental Association held its dinner and scientific meeting at the Hotel Statler, Sept. 10. Dr. Francis E. Fronczak and Alderman Staples were guests of honor. The matter of dental inspection of school children, which was voted down by the common council last spring, was brought up anew and it is hoped that this important reform will be carried through.

The National Association for the Study of Pellagra holds its second triennial meeting at Columbia, S. C., Oct. 3 and 4.

42 papers are scheduled beside as many more not designated by title. We cannot refrain from suggesting that, for any meeting, the incisive should not so far exceed the molar capacity.

---

The American Association for the Study and Prevention of Infant Mortality will hold its third annual meeting at Cleveland, Oct. 2-5. As is obvious from the duration of the meeting this association includes educators and sociologists as well as physicians and will discuss various phases of infant welfare not technically medical.

---

The Eighth District Branch of the Medical Society of the State of New York met in Buffalo, Sept. 24 and 25. The afternoon session on the first day was devoted to business, President's address by Dr. Henry A. Eastman of Jamestown, Remarks on Some of the Needs of the State Society by Dr. John F. W. Whitbeck of Rochester, and scientific papers. The morning of the second day was devoted to ambulatory and surgical clinics by Buffalo men. A dinner and luncheon were served at the University Club. The meetings were held in Alumni Hall of the University of Buffalo. Our readers will appreciate the fact that meetings occurring later than the 20th of any month cannot be adequately reported till the second month, when, often, the proceedings are too well known to be news.

---

The Chemung Co. Medical Society held its first fall meeting at Elmira, Sept. 17. Dr. Anna Stuart read a paper on the N. Y. City Health Dept. and Dr. C. R. Jennings on the Treatment of Inoperable Carcinoma.

---

The Homoeopathic Medical Society of the State of New York will meet at the Lafayette Hotel, Buffalo, Oct. 8 and 9, under the presidency of Dr. Willis B. Gifford. Various entertainments have been planned in addition to the scientific program, the entertainment committee consisting of Drs. Critchlow, Maycock, Moseley, Pinkerton and Case.

